# Circulation of *Anaplasma phagocytophilum* among invasive and native carnivore species living in sympatry in Poland

**DOI:** 10.1186/s13071-023-05996-7

**Published:** 2023-10-18

**Authors:** Paulina Maria Lesiczka, Izabella Myśliwy, Katarzyna Buńkowska-Gawlik, David Modrý, Kristýna Hrazdilová, Joanna Hildebrand, Agnieszka Perec-Matysiak

**Affiliations:** 1 Department of Veterinary Sciences, Faculty of Agrobiology, Food and Natural Resources, Czech University of Life Sciences Prague, Prague, Czech Republic; 2 Department of Parasitology, Faculty of Biological Sciences, University of Wrocław, Wrocław, Poland; 3 Biology Centre, Institute of Parasitology, Czech Academy of Sciences, České Budějovice, Czech Republic; 4 Department of Botany and Zoology, Faculty of Science, Masaryk University, Brno, Czech Republic; 5 Faculty of Medicine in Pilsen, Biomedical Center, Pilsen, Czech Republic; 6 Department of Chemistry and Biochemistry, Mendel University, Brno, Czech Republic

**Keywords:** *Anaplasma phagocytophilum*, Carnivores, *Meles meles*, *Martes* spp., *Nyctereutes procyonides*, *Procyon lotor*, *Vulpes vulpes*, Invasive species

## Abstract

**Background:**

*Anaplasma phagocytophilum* is characterized by a worldwide distribution and distinguished from other Anaplasmataceae by the broadest range of mammalian hosts and high genetic diversity. The role carnivores play in the life cycle of *A. phagocytophilum* in Europe is uncertain. Currently, only the red fox is considered a suitable reservoir host. In this study, we focused on native and invasive medium-sized carnivore species that live in sympatry and represent the most abundant species of wild carnivores in Poland.

**Methods:**

A total of 275 individual spleen samples from six carnivore species (*Vulpes vulpes, Meles meles, Procyon lotor, Nyctereutes procyonoides* and *Martes* spp.) were screened combining nested PCR and sequencing for *A. phagocytophilum* targeting a partial *groEL* gene with subsequent phylogenetic analysis inferred by the maximum likelihood method.

**Results:**

The DNA of *A. phagocytophilum* was detected in 16 of 275 individuals (5.8%). Eight unique genetic variants of *A. phagocytophilum* were obtained. All detected haplotypes clustered in the clade representing European ecotype I. Three variants belonged to the subclade with European human cases together with strains from dogs, foxes, cats, and wild boars.

**Conclusions:**

While carnivores might have a restricted role in the dissemination of *A. phagocytophilum* due to their relatively low to moderate infection rates, they hold significance as hosts for ticks. Consequently, they could contribute to the transmission of tick-borne infections to humans indirectly, primarily through tick infection. This underscores the potential risk of urbanization for the *A. phagocytophilum* life cycle, further emphasizing the need for comprehensive understanding of its ecological dynamics﻿.

**Graphical Abstract:**

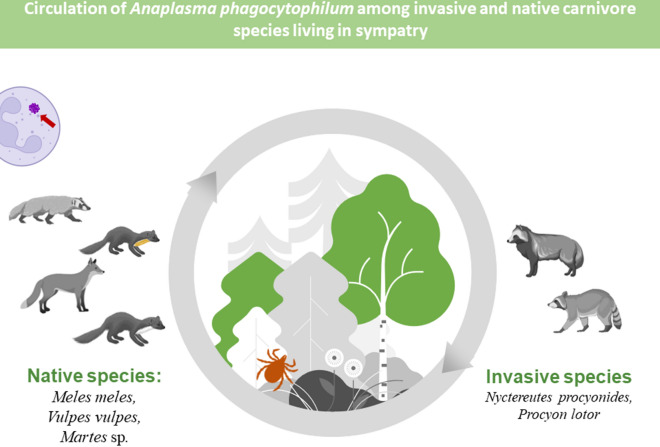

## Background

*Anaplasma phagocytophilum* is a gram-negative alpha-proteobacterium infecting neutrophils. It is characterized by a broad distribution [1, 2] and distinguished from other Anaplasmataceae bacteria by the widest range of mammalian hosts and high genetic diversity [3]. Based on studies focused on ecology and genetic diversity, the species of *A. phagocytophilum* consists of at least four major ecotypes, of which only ecotype I has been proven to infect humans in Europe so far [4, 5]. The main hosts of ecotype I are ungulates [6, 7], dogs, cats, horses [8–10], and various wild mammals in urban or suburban areas, such as red foxes (*Vulpes vulpes*) [11–13], hedgehogs (*Erinaceus* sp.) [14–17], and wild boars (*Sus scrofa*) [18–20]. European ecotype I of *A. phagocytophilum* is mainly transmitted by the tick *Ixodes ricinus*, characterized by low host specificity [21, 22]. To some extent, the nest-dwelling *I. hexagonus*, which is known to parasitize hedgehogs, red foxes, and European badgers, is involved in the circulation of ecotype I of *A. phagocytophilum* [23, 24]. Occasionally, species from other tick genera tested positive for the presence of *A. phagocytophilum* DNA; however, their significance is currently unknown [25–28].

In Europe, *A. phagocytophilum* has been detected by molecular methods in wild carnivores from six families: Canidae, Ursidae, Mustelidae (Caniformia), Felidae, Procyonidae, and Viverridae (Feliformia) [3, 29–31]. Although the role of wild carnivores as reservoir hosts for this pathogen in Europe is uncertain, some species such as raccoon dogs and red foxes are capable of transmitting *A. phagocytophilum* in nature. In this study, we focused on native and invasive medium-sized carnivore species living in sympatry and representing the most abundant species of wild carnivores in Poland. Thus, the objectives of this study were to understand the genetic diversity of *A. phagocytophilum* in wild invasive and native carnivores with overlapping ranges and to investigate the possibility of cross-species transmission of genetic variants (including zoonotic ones) of *A. phagocytophilum* between these species.

## Materials and methods

### Study area and sampling

The carcasses of red fox, raccoon dog, raccoon, badger, and marten were collected in the forestry of Ruszów (51° 24′ 00.1″ N 15° 10′ 12.2″ E) in the Lower Silesia County in Poland during the predator control, which was part of the program for the reintroduction of capercaillie (*Tetrao urogallus*) in the Lower Silesian Forest (project LIFE11 NAT /PL/428) in the years 2017–2019. All carcasses were frozen and transported to the Department of Parasitology, University of Wrocław. A total of 275 individual spleen samples from six carnivore species, red fox (*V. vulpes*) (*n* = 48), raccoon dog (*Nyctereutes procyonoides*) (*n* = 50), raccoon (*Procyon lotor*) (*n* = 42), badger (*Meles meles)* (﻿*n* = 51), beech marten (*Martes foina*) ﻿(*n* = ﻿57), and European pine marten (*Martes martes*) ﻿(*n* = 27) were collected during necropsy. All samples were kept at − 20 °C until further DNA isolation procedures﻿.

### DNA extraction, PCR protocols and sequencing

DNA was extracted from 10 mg of spleen using the commercial GeneMatrix Bio-Trace DNA Purification Kit (EURx, Poland) according to the manufacturer’s instructions. PCRs for detection of *A. phagocytophilum* were performed using 2 × PCRBIO Taq Mix Red (PCR Biosystems, UK). To determine the *groEL* ecotype of *A. phagocytophilum*, 1297 bp fragments of the *groESL* operon or (in the case of a missing amplicon) 407 bp of the *groEL* gene were amplified by nested PCR as previously described [18]. To distinguish two marten species (*M. martes and M. foina*) the rapid PCR–RFLP method described by Vercillo et al. [32] was used.

Amplicons were separated by electrophoresis in a 1.5% agarose gel stained with Midori Green Advance (Nippon Genetics Europe, Germany) gel stain and visualized under UV light. All PCR products of the expected size were excised from the agarose gels, purified, and sequenced in both directions using the amplification primers. Sequencing was performed by Macrogen Capillary Sequencing Services (Macrogen Europe, the Netherlands). The sequences obtained were processed using the Geneious 11.1.4 software [33] and compared with those available in the GenBank™ dataset by Basic Local Alignment Tool (BLAST).

### Phylogenetic analysis

The phylogeny of *A. phagocytophilum* was constructed using eight unique *groEL* haplotypes detected in this study along with 65 sequences from GenBank, representing four ecotypes described by Jahfari et al. [4] and a sequence from *Anaplasma platys* used as outgroup. Due to unequal sequence lengths, the alignment was calculated in two steps using the MAFFT algorithm ‘Auto’ strategy for sequences > 1000 nt and the –add function for implementing sequences < 1000 nt in the alignment with final length of 1402 nt. The phylogenetic tree was inferred by the maximum likelihood method by IQTREE 1.6.5 [34]. The best-fit evolution model was selected based on the Bayesian information criterion (BIC) computed by implemented ModelFinder [35]. Branch supports were assessed by the ultrafast bootstrap (UFBoot) approximation [36] and by the SH-like approximate likelihood ratio test (SH-aLRT) [37]. Trees were visualized and edited in FigTree v1.4.1 and Inkscape 0.91.

## Results

**Fig. 1:**
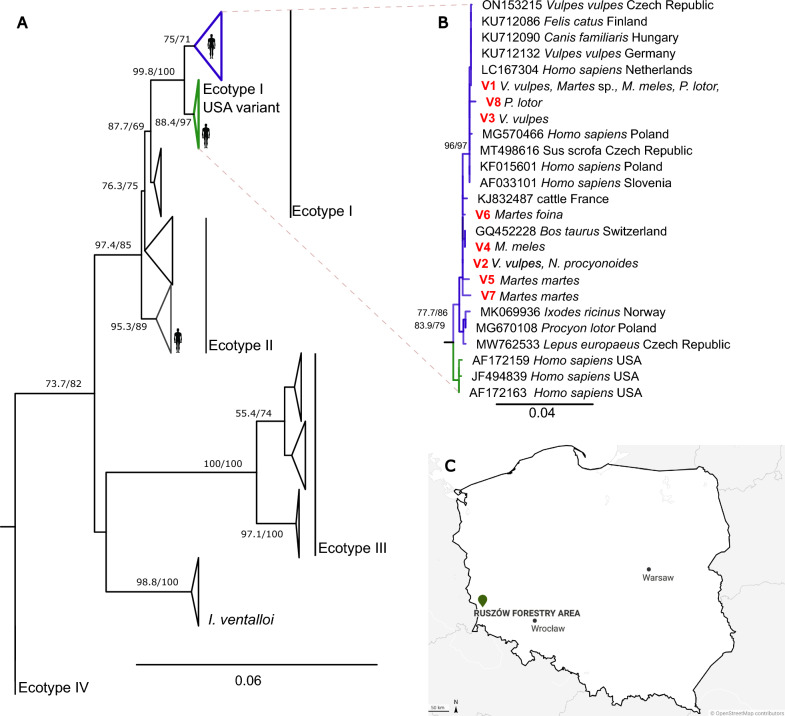
**A** Schematic representation of the maximum likelihood phylogenetic tree based on the *groEL* gene sequences of *Anaplasma phagocytophilum* representing all ecotypes. The highlighted clade representing Ecotype I is displayed in detail; bootstrap values (SH-aLRT/UFB) above the 70/70 threshold are displayed; sequence of *Anaplasma platys* used as an outgroup is not shown. **B** Detailed view of the clade representing the Ecotype I/Cluster I; sequences acquired from the GenBank database are marked by their accession number, host, and country of origin. Sequences from this study are highlighted in red and marked by the number of a respective variant. The scale bar indicates the number of nucleotide substitutions per site. **C** Map of Poland with a detailed locality of Ruszów Forestry sampling area

The DNA of *A. phagocytophilum* was detected in 16 of 275 individuals (5.8%). The number of positive animals per species ranged from one (2%) in raccoon dog to five (8.8%) in beech marten (Table 1). Three long (> 1000 nt) and 13 short (300–400 nt) sequences of the *groEL* gene representing 8 unique genetic variants were obtained. The major genetic variant V1 was detected in seven samples derived from four martens and a single European badger, red fox, and raccoon, respectively. Two other variants, V2 and V3, were detected in two animals each. Variant V2 was found in red fox and racoon dog, and variant V3 was detected in samples from red foxes only. The remaining variants V4–V8 were detected in one sample each from three martens, one badger, and one raccoon (Table 1). The representative sequences were submitted to the GenBank under the accession number OR167090-OR167101. In phylogenetic analyses (Fig. 1), all detected haplotypes clustered in the largest clade representing European ecotype I [4], which is closely related to isolates from the USA and forms cluster I [5]. Three variants (V1, V3, and V8) belonged to the subclade with European human cases and strains from dogs, foxes, cats, and wild boars. The remaining five variants were distributed among strains isolated from *I. ricinus*, European hares, carnivores, and sequences obtained from ungulates.

**Table 1:** The prevalence of *Anaplasma phagocytophilum* among invasive and native carnivore species living in sympatry in Poland

Species	Total number of animals	No. of positive animals/prevalence	Genetic variant^a^
Red fox (*Vulpes vulpes)*	48	3/6.2%	V1, V2, V3
Raccoon (*Procyon lotor*)	42	2/4.7%	V1, V8
Raccoon *dog* (*Nyctereutes procyonoides*)	50	1/2%	V2
Beech marten (*Martes foina*)	57	5/8.8%	V1, V6
European pine marten (*Martes martes*)	27	3/11%	V1, V5, V7
Meles meles (*Meles meles*)	51	2/3.9%	V1, V4

^a^All genetic variants detected in this study belong to ecotype-I [4]

## Discussion

The persistence and transmission of tick-borne pathogens in ecosystems relies upon abundance of susceptible reservoir hosts and their infestation by permissive tick species. Studies on European strains of *A. phagocytophilum* have shown that a wide range of animal species are involved in the circulation of this pathogen in different ecological niches [38]. Among all *Anaplasma* spp*., A. phagocytophilum* represents an assemblage with enormous genetic diversity. Clarifying which host species harbor specific strains of *Anaplasma* is important for understanding pathogen dynamics and for developing measures to reduce disease burden [39]. The role of carnivores in the ecoepidemiology of *A. phagocytophilum* is not well understood. While several wild carnivores have been implicated as possible reservoirs for *A. phagocytophilum* in the US, only the red fox has been considered a suitable host in Europe [12, 29, 30, 40–42]. Carnivores such as badgers and martens are often overlooked in studies. This information gap also affects invasive species such as raccoons and raccoon dogs, which were intentionally introduced to Europe and later spread through the continent [43]. In our study, we have shown that both native (foxes, badgers, martens) and invasive (raccoons) carnivores living in sympatry in a forest biotope are involved in the circulation of *A. phagocytophilum* with zoonotic potential, finding the genetic variant V1 in all examined species except raccoon dogs (Table 1, Fig. 1).

The red fox is the most widespread free-living predator in the world [44], and its role as a host for *A. phagocytophilum* is well documented [45, 46]. In Poland, *A. phagocytophilum* has been detected in foxes with prevalence ranging from 2.7% in the central part of the country [11] to 34.5% in the northeastern regions [31]. In our study, 6.2% of animals tested positive for this pathogen, which is consistent with the general trend observed for *Anaplasma* infections in the European fox population and supports foxes as a reservoir of *A. phagocytophilum.*

Only a few previous studies have focused on the role of mustelids in the circulation of *A. phagocytophilum*. In this study, 3.9% of badgers and 9.5% of martens were positive for *A. phagocytophilum* DNA. Analyses focused on badgers and martens from eastern and northern Poland detected the DNA of *A. phagocytophilum* in 18.7% and 41.7% of animals, respectively [31]. For comparison, the number of positive badgers from Spain and The Netherlands did not exceed 2% [39, 47]. Data on *A. phagocytophilum* in European marten populations are sparse. To our knowledge, this pathogen has been detected so far in a beech marten from Romania [28] and a pine marten from Hungary [45] in which ecotype I was recognized [4]. In addition, in mustelids from The Netherlands tested by quantitative polymerase chain reaction (qPCR) for several Tick Borne Pathogens (TBPs), *A. phagocytophilum* was detected in beech martens (1.5%), European badgers (1.8%), European polecats (*Mustela putorius*) (4.9%), and pine martens (22%) [39]. The observed discrepancies in overall prevalence are likely due to the specific environmental conditions under which each study was conducted, affecting tick occurrence and density. The type of tissue and molecular method used to detect pathogens may also explain the differences in results [17]. The results of our study indicate that martens are significantly more susceptible to *Anaplasma* infection, with a consistent increase in prevalence observed in these predators in all cited studies. The differences in distribution patterns between the two species (the pine marten has a patchy, fragmented ecogeographic distribution restricted to a narrow ecological niche, whereas the beech marten has a continuous distribution across a wide range of natural, semi-natural, and even urban habitats) may have implications for the ecoepidemiology of *A. phagocytophilum*, particularly in the context of rapid landscape change and intense urbanization processes. Nevertheless, the current lack of comprehensive studies makes it difficult to fully elucidate the relationships. Determining whether martens have exclusive host/reservoir competence for *Anaplasma*, Anaplasmataceae, or other tick-borne pathogens is a complex task that requires further investigation. Invasive carnivore species, such as raccoons and raccoon dogs, are potential reservoirs for numerous TBPs [43, 48], and we also found *A. phagocytophilum* in 2% of raccoon dogs and 4.7% of raccoon dogs. The prevalence of *A. phagocytophilum* previously observed in raccoon dogs from Poland (35.3%) [31] was higher than in Germany (23%) [47]. Kjær and colleagues reported a high clustering of *A. phagocytophilum*-positive ticks on individual raccoon dogs in Denmark [49]. Raccoons from Austria, the Czech Republic, Germany, and Poland [50, 51] were tested for the presence of *A. phagocytophilum* DNA, but the pathogen was found in only one raccoon from the latest study [43]. Our results show that raccoons are adapted to carry European variants of *A. phagocytophilum*. Due to their synanthropic nature and frequent use of tree holes and burrows of other animal species, raccoons can be infested with both questing and endophilic ticks, potentially bridging the enzootic cycles of *A. phagocytophilum*. Regarding the epidemiological impact of raccoons and raccoon dogs, these invasive species should be monitored for their possible involvement in the spread of *A. phagocytophilum* in different geographic regions [51].

In recent years, awareness of role of wildlife in TBPs and the possible impact on livestock, humans, and their pets has increased [52]. Knowledge of potential reservoir hosts and their ticks is necessary to develop effective surveillance and management measures for disease outbreaks and parasite cycles in wildlife [53]. Nidicolous ticks such as *Ixodes hexagonus*, which are commonly found on foxes and have been detected on mustelids [39, 54], deserve future attention as they may play a role as vectors for zoonotic variants of *A. phagocytophilum*. In addition, high population densities of predator populations are possible in European landscapes with heterogeneous habitat structure, leading to shared territories among red foxes, raccoons, and raccoon dogs [55–57], favoring the transmission of vectors and pathogens. The increasing distribution and numbers of foxes in urban and suburban areas make this species a bridging species between natural ecosystems and anthropogenic landscapes [39].

## Conclusions

While carnivores might have a restricted role in the dissemination of *A. phagocytophilum* due to their relatively low to moderate infection rates, they hold significance as hosts for ticks. Consequently, they could contribute to the transmission of tick-borne infections to humans indirectly, primarily through tick infection. This underscores the potential risk of urbanization for the *A. phagocytophilum* life cycle, further emphasizing the need for comprehensive understanding and management of its ecological dynamics.

## Data Availability

Data will be made available on request.
